# Biodegradable Poly(Amino Ester) with Aromatic Backbone as Efficient Nonviral Gene Delivery Vectors

**DOI:** 10.3390/molecules22040566

**Published:** 2017-03-31

**Authors:** Qiang Liu, Rong-Chuan Su, Wen-Jing Yi, Zhi-Gang Zhao

**Affiliations:** College of Chemistry and Environmental Protection Engineering, Southwest Minzu University, Chengdu 610041, China; lqchem@163.com (Q.L.); Surongcc@163.com (R.-C.S.)

**Keywords:** gene delivery, non-viral vectors, aromatic backbone, cationic polymer, biodegradable

## Abstract

The development of gene delivery vectors with high efficiency and biocompatibility is one of the critical points of gene therapy. Two biodegradable poly(amino ester)s were synthesized via ring-opening polymerization between low molecular weight (LMW) PEI and diepoxide. The molecular weights of poly(amino ester)s were measured by GPC. Agarose gel retardation assays showed that these materials have good DNA-binding ability and can retard the electrophoretic mobility of plasmid DNA (pDNA) at a weight ratio of 1. The formed polyplexes have proper sizes of around 200 nm and zeta-potential values of about 30–40 mV for cellular uptake. In vitro experiments revealed that polymer **P2** gave higher transfection efficiency than PEI 25 KDa and Lipofectamine 2000 with less toxicity, especially in 293 cells. Results demonstrate that such a type of degradable poly(amino ester) may serve as a promising non-viral gene vector.

## 1. Introduction

Gene therapy affords a promising way to treat congenital and acquired diseases [1,2]. Safe and efficient delivery of genetic material into target cells remains a challenging aspect of gene therapy [3]. Although viral vectors are considered to have high gene transfer efficiency, their clinical applications have been greatly restricted by a number of key issues such as immunogenicity, insertional mutagenesis, oncogenic effects, and toxicity [4,5]. As safer alternatives, non-viral gene vectors have received increasing attention for their easy preparation and modification [6,7,8]. Numerous cationic polymers have been developed for fabricating non-viral carrier systems, e.g., polyethylenimine (PEI) [9,10], poly(l-lysine) (PLL) [11,12], polyamidoamine dendrimers (PAMAM) [13,14], and chitosan [15,16] etc. 

Among these polymeric polymers, PEI is the most widely studied gene carrier [17]. High molecular weight (HMW) PEI, especially with the Mw of 25 KDa, has relatively higher transfection efficiency (TE) both in vitro and in vivo [18]. This made it the “golden standard” for the design of six novel polymeric vectors [19,20]. In addition, a linear form with an average molecular weight of 22 kDa has also been described with high transfection activity [21,22]. However, the high charge density and lack of biodegradability contribute significantly to its evident cytotoxicity in HMW PEI-mediated gene delivery [23,24,25]. Compared to the HMW PEI, the low molecular weight (LMW) PEI demonstrates much lower toxicity, yet almost no transfection activity [17,26,27]. Therefore, to develop novel PEI-based vectors with both high TE and low cytotoxicity is of great importance. Linking LMW PEI to some special groups, such as ester, phosphoester, ketal, disulfide, carbamate, amide, etc. to form a biodegradable cationic polymer is an effective strategy [28,29,30,31,32]. These degradable PEIs have shown significantly enhanced transfection activity compared to the parent LMW PEI and retain their biocompatibility due to groups or bonds degradation in cellular environments.

Our group recently revealed that cationic polymers with rigid aromatic structure in the backbone showed significantly increased TE [33,34]. The aromatic moiety might play an important role in enhancing the DNA condensation ability of cationic polymers by π-stacking interaction with DNA, which contribute to more stable polyplexes and favor cellular uptake. Additionally, we also found that ring-opening polymerization from bis-epoxides is an effective synthetic approach toward gene delivery materials with high biological activity [35,36,37]. The newly formed hydroxyls and oxygen atoms in the backbones could effectively enhance TE and serum-tolerance similar to PEG structure. Herein in this report, we synthesized two kinds of LMW PEI-based biodegradable poly(amino ester) with aromatic backbones through the epoxide ring-opening polymerization. We characterized the molecular weights and structures of the novel polymers. Their gene delivery properties were also systematically investigated by acid-base titration, DNA-binding assay, cytotoxicity assay, and in vitro cell transfection studies.

## 2. Results and Discussion

### 2.1. Synthesis and Characterization of Target Polymers

**Scheme 1:**
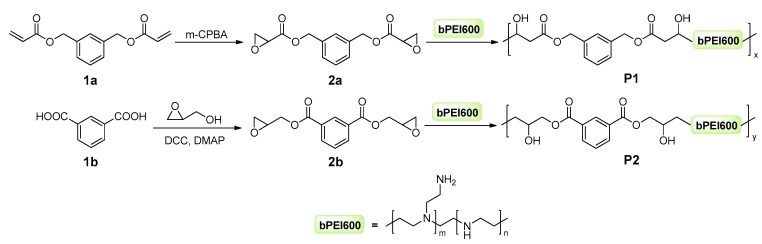
Synthetic route of the target polymers.

The aim of this study was to rationally design and develop a novel gene delivery vector based on degradable poly(amino ester). As shown in Scheme 1, the diglycidyl ethers **2a**, **2b** as linking moiety were separately prepared by oxidation and ester condensation reaction. Subsequently, ring-opening polymerization was simply processed by mixing **2a** or **2b** and PEI 600 Da with an appropriate amount ratio (mole ratio = 1:1 of monomer) under reflux temperature. The crude product was recrystallized 3 times with anhydrous DCM and cyclohexane to ensure their polydispersity. The structural difference between **P1** and **P2** is the orientation of the ester group, helping us to elucidate the structure-activity relationships (SAR). Additionally, it is expected that the presence of different types of amino groups in the LWM PEI cationic moiety could efficiently improve gene delivery efficiency. The primary amines the polymer bears are capable of condensing DNA into complexes through electrostatic interaction, while the secondary and tertiary amines provide the polycations with high buffer capacity, which can facilitate the endosomal escape [6,7,17]

Gel permeation chromatography (GPC) was used to measure the molecular weights of target compounds. Results show that the cationic polymer **P2** (*M_n_* = 24,550) has a larger molecular weight than **P1** (*M_n_* = 17,561). As is well-known, the degradation of polymers makes them easy to be eliminated through the excretion pathway in vivo [38]. Further, the degradability of the polymeric material was also studied by GPC. In 48 observation hours, the *M_n_* of **P2** was finally decreased to 20,218 Da (Figure S1 in Supplementary Materials). Results affirmed the degradation of the target polymers in physiological solution.

### 2.2. Buffer Capacity

It was assumed that the buffering capacity of cationic polymers facilitates the escape of polymer/DNA complexes from the endosome, and to promote TE, which was known as the “proton-sponge effect” [39]. The buffering capacities of polymers, expressed as the percentage of amino nitrogen that could be protonated in the endosome pH range of 7.4–5.1, were examined by acid-base titration. The results are shown in Table 1 and Figure S2 in the supplementary information. The target polymers, especially **P2**, definitely showed a higher buffering capacity (28.37%) than “golden standard” PEI 25 KDa (18.09%) and PEI 600 Da (11.20%), indicating their excellent buffering capacities. The lower buffering capability of **P1** (25.85%) may be ascribed to its lower molecular weight.

### 2.3. The Formation and Properties of Polymer/DNA Polyplexes

**Figure 1:**
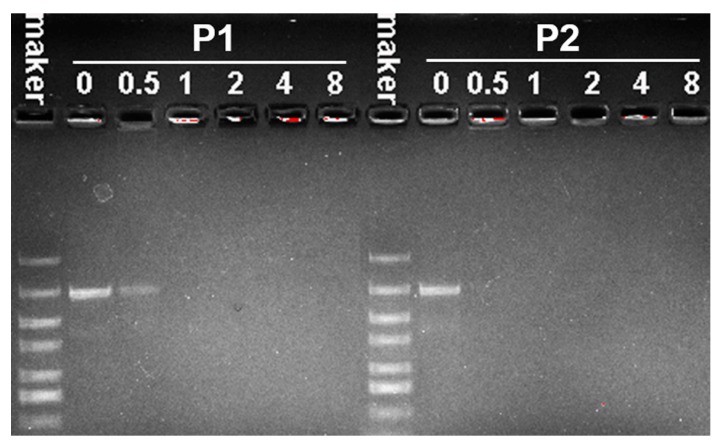
Agarose gel electrophoresis of cationic polymers complexed with DNA to form polyplexes at different weight ratios: 0.5 (N/P = 2.5), 1 (N/P = 4.9), 2 (N/P = 9.8), 4 (N/P = 19.6), and 6 (N/P = 29.4).

Good DNA condensation capability of polycations is a prerequisite for cationic gene carriers. Electrostatic interaction is the predominant mode of polycations–nucleic acid interactions. Besides, site- and structure-specific binding has also been recognized [40,41,42]. The formation of a polyplex can reduce the electrostatic repulsion between DNA and the cell surface, and can protect DNA against enzymatic degradation by nucleases in cytoplasm or serum [43]. Agarose gel electrophoresis was performed to investigate the binding capability of **P1** and **P2** toward plasmid DNA at various weight ratios (polymer/DNA, *w*/*w*). As shown in Figure 1, full retardation of plasmid DNA was observed from the *w*/*w* ratio of 1. The result demonstrated that both polymers can effectively bind DNA at a relatively low dosage with excellent DNA binding capacity.

**Figure 2:**
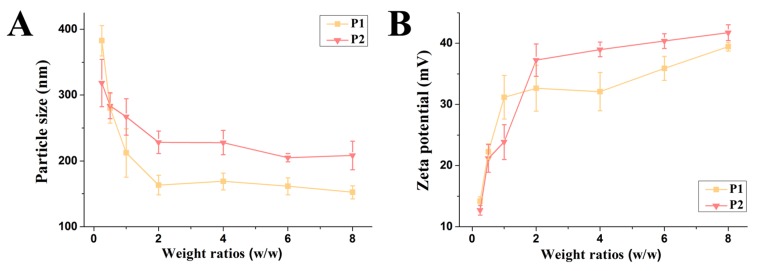
Mean particle size (**A**) and zeta potential (**B**) of polyplexes obtained at various weight ratios by dynamic light scattering (DLS). Data represent mean ± SD (*n* = 3).

The proper particle size and positive surface charge of polyplexes are also important for cellular uptake and efficient gene delivery. As reported in the literature, the particles with mean diameters between 54 and 625 nm are most liable to endocytosis by cells [44]. These properties of complexes formed at various weight ratios ranging from 0.25 to 8, and were measured by dynamic light scattering (DLS). Results in Figure 2A reveal that, with the increase of *w*/*w* ratio (>2), the sizes of nanoparticles plateaued with mean diameters of 150–230 nm. On the other hand, a cationic surface charge is beneficial because it aids in the electrostatic interaction between polyplexes and negatively charged cellular membranes, leading to efficient intracellular uptake. As shown in Figure 2B, zeta-potential of the polyplexes increased along with the increase of *w*/*w* ratio, and the surface charge was observed about +31–41 mV above *w*/*w* ratio of 2. The lower zeta-potential of **P1** might be attributed to the closer oxygen-rich structure with amino groups, resulting in the screening of positive charges.

### 2.4. In Vitro Transfection 

**Figure 3:**
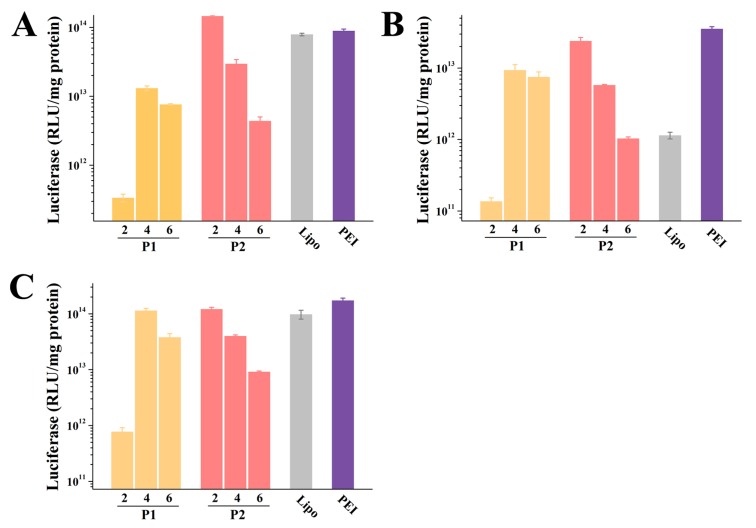
Luciferase gene expression transfected by polyplexes at different weight ratios: 2 (N/P = 9.8), 4 (N/P = 19.6), and 6 (N/P = 29.4) in comparison with Lipofectamine 2000 and 25 KDa PEI (*w*/*w* = 1.4, N/P = 10) in human embryonic kidney cell lines (HEK 293) (**A**); human cervix carcinoma cell lines (HeLa) (**B**), and human hepatoma cell lines (HepG2) (**C**) cells in the absence of serum. Data represent mean ± SD (*n* = 3).

Luciferase reporter gene was used to quantitatively assess the in vitro TEs of the new polyplexes in several cell lines, including human embryonic kidney cell lines (HEK 293), human cervix carcinoma cell lines (HeLa), and human hepatoma cell lines (HepG2). Figure 3 exhibits the TEs of **P1** and **P2** at various *w*/*w* ratios as the relative fluorescence intensity per mg protein (RLU/mg protein), and PEI 25 KDa and Lipofectamine 2000 were used for comparison. The new cationic polymer **P2** showed much higher TEs in all tested cell lines, and the TEs were greatly dependent on the cell type. As shown in Figure 3A, the complexes made by **P2** shows 1.63-fold higher TE than PEI 25 KDa, and 1.85-fold higher TE than Lipofectamine 2000, toward HEK 293 cells at the optimized *w*/*w* ratio of 4. Meanwhile, **P2** also gave comparable TEs to PEI 25 KDa and Lipofectamine 2000 in tumor cells (Figure 3B,C). Under the same condition, **P1** showed much lower TEs than **P2**. For the ester-contained polymers **P1** and **P2**, such subtle differences in their structure led to completely different TEs. We speculate that **P2**, with higher surface charge and better buffering capacity, may promote more efficient cellular uptake and endosome escape of the complexes. The detailed transfection mechanisms leading to such differences are under further investigation.

**Figure 4:**
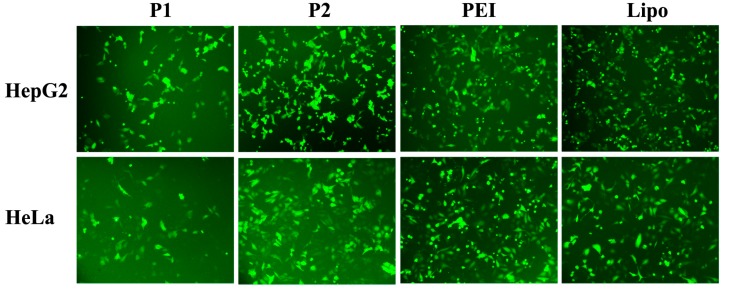
Fluorescence microscope image of pEGFP-transfected HepG2 and HeLa cells in the absence of serum at optimal weight ratio.

To directly visualize the infected cells expressing the pEGFP-Nl reporter gene, polymers **P1** and **P2** mediated enhanced green fluorescent protein (eGFP) expression in HepG2 and HeLa cells was observed by an inverted fluorescent microscope. The weight ratios were used according to the optimal results from luciferase assays, and the images are shown in Figure 4. Similar to the results in luciferase assay, polymer **P1** gave much better GFP expression than the others involving controls, and much higher green fluorescence density could be observed.

**Figure 5:**
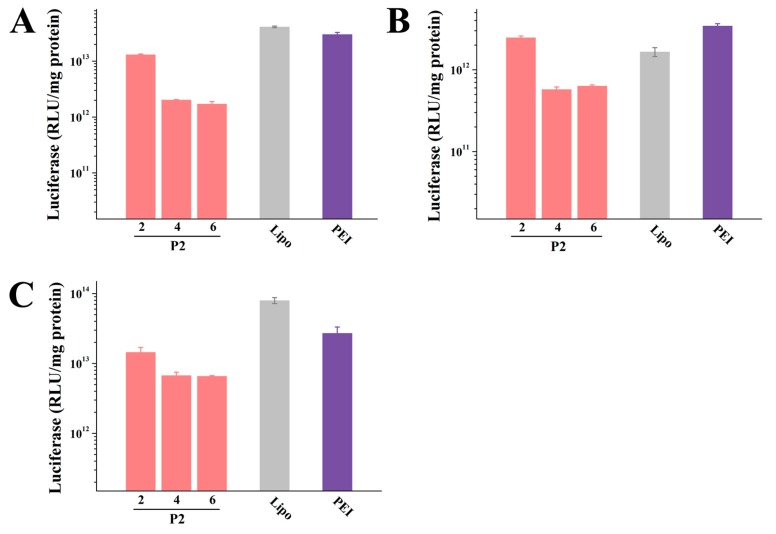
Transfection by **P2** at various weight ratios: 2 (N/P = 9.8), 4 (N/P = 19.6), and 6 (N/P = 29.4) in HEK 293 (**A**); HeLa (**B**) and HepG2 (**C**) cells in the presence of 10% serum. Lipofectamine 2000 and PEI 25 KDa (*w*/*w* = 1.4, N/P = 10) were used as controls. Data represent mean ± SD (*n* = 3).

Furthermore, to estimate the in vivo transfection potential, we studied the serum-present gene delivery ability of cationic polypexes as a fundamental predictive model. The serum-triggered transfection inhibition was believed to have a strong association with the non-specific interaction between complexes and negatively charged proteins [45,46]. For the higher TEs of **P2** in preceding serum-free studies, this polymer was subsequently chosen to study its transfection behavior in all the cell lines in the presence of 10% serum. As shown in Figure 5, the TEs of **P2**/DNA polyplexes were lower than that obtained in the absence of serum and the controls PEI 25 KDa and Lipofectamine 2000. Further studies to modify of these types of polymers to improve the TE are now in progress.

## 3. Cytotoxicity

**Figure 6:**
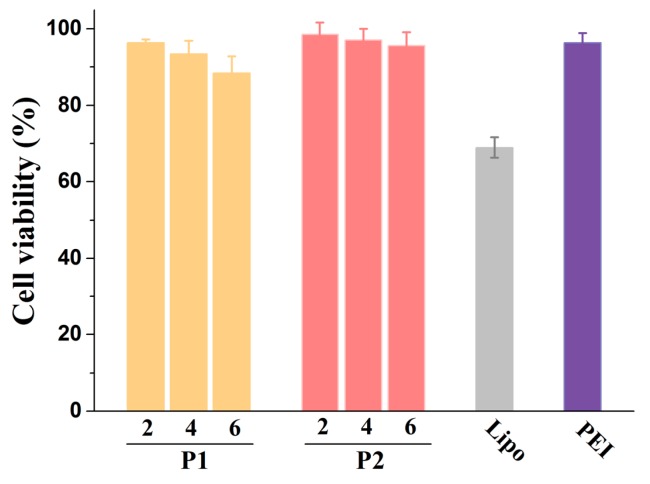
Cytotoxicity of polyplexes at different weight ratios toward HepG2 cells. Lipofectamine 2000 and PEI 25 KDa were used as controls (*w*/*w* = 1.4). Data represent mean ± SD (*n* = 3).

The negatively-charged cellular and blood components would interact with cationic polyplexes, leading to inherent toxicity [47]. Therefore, cytotoxicity is a crucial factor in assessing biosafety for polymeric gene carriers. A successful delivery system should have high TE and low toxicity. The cell viability of the target polymer/DNA complexes was evaluated in HepG2 cells by CCK-8 assays, using PEI 25 KDa and Lipofectamine 2000 as controls. It should be noted that the weight ratio for CCK-8 assay was the same with that used in the transfection experiment. As shown in Figure 6, the cytotoxicity of polyplexes gradually increased with increasing of the weight ratios, due to the presence of higher cationic charge density. At a low weight ratio (=2), almost no cytotoxicity was observed, and the cell viabilities of studied polyplexes (especially **P1**) were also higher than that involving PEI 25 KDa and Lipofectamine 2000, even at higher weight ratios. Comparing with the structure of PEI 25 KDa, we believe that the lower cytotoxicity may be attributed to the newly formed hydroxyl groups and biodegradable ester linkages, which can benefit the biocompatibility of the polymeric materials used for gene transfection [24,37]. It is also proved that the ring-opening polymerization from bis-epoxides is an effective synthetic approach toward materials with low cytotoxicity.

## 4. Experimental Section

### 4.1. Materials and Methods

All chemicals and reagents were obtained commercially and were used as received. Anhydrous ethanol and dichloromethane (DCM) were dried and purified under nitrogen by using standard methods and were distilled immediately before use. (2-nitro-1,3-phenylene)bis(methylene) diacrylate (**1a**) was prepared according to the literature [48]. LMW PEI (branched, average molecular weight 600 Da, 99%), branched PEI 25 KDa and MTT (3-(4,5-dimethylthiazol-2-yl)-2,5-diphenyltetrazolium bromide) were purchased from Sigma-Aldrich (St. Louis, MO, USA). The plasmids used in the study were pGL-3 (Promega, Madison, WI, USA) coding for luciferase DNA and pEGFP-N1 (Clontech, Palo Alto, CA, USA) coding for EGFP DNA. The Dulbecco’s Modified Eagle’s Medium (DMEM) and fetal bovine serum were purchased from Invitrogen Corp. MicroBCA protein assay kit was obtained from Pierce (Rockford, IL, USA). Luciferase assay kit was purchased from Promega (Madison, WI, USA). Endotoxin free plasmid purification kit was purchased from TIANGEN (Beijing, China). HEK 293 cells, HeLa cells, and HepG2 cells were purchased from Shanghai Institute of Biochemistry and Cell Biology, Chinese Academy of Sciences. All other reagents used in the synthesis, if not specified, were obtained from Sigma-Aldrich Co. and used without further purification.

The ^1^H-NMR and ^13^C-NMR spectra were obtained on a Varian INOVA-400 spectrometer. CDCl_3_ or D_2_O was used as the solvent, and TMS was used as the internal reference. The MS (ESI) spectra data were recorded with a Finnigan LCQDECA and with a Bruker Daltonics BioTOF mass spectrometer (Bruker Daltonics Inc., Billerica, MA, USA). The molecular weight of polymers was determined by gel permeation chromatography (GPC) 515 pump, Waters 2410 Refractive Index Detector (Waters Corp, Milford, MA, USA), 25 °C, incorporating Shodex columns OHPAK KB-803, (Showa Denko Corp., Tokyo, Japan). A filtered mixture of 0.5 mol·L^−1^ HAc/NaAc buffer was used as the mobile phase, with a flow rate of 0.5 mL·min^−1^. Molecular weights were calculated against polyethylene glycol standards, with average molecular weights ranging from 900 to 80,000.

### 4.2. General Method for Preparation of Biodegradable Poly(Amino Ester) with Aromatic Backbone

#### 4.2.1. Synthesis and Characterization Linker of **2a**

(2-nitro-1,3-phenylene)bis(methylene) diacrylate (**1a**) (492 mg, 2 mmol) was oxidized by meta-chloroperoxybenzoic acid (*m*-CPBA, 1.73 g, 8 mmol, 80% purity) in 30 mL DCM. The mixture was refluxed at 55 °C for 8 h. After the reaction, the solution was washed with sat. NaHCO_3_ (2 × 50 mL), and brine (2 × 50 mL). The organic layer was dried over MgSO_4_, and concentrated under reduced pressure. The residue was purified by silica gel column chromatography (silica, ethyl acetate/petroleum ether: *v*/*v* = 1/4) to give product **2a** (pale-yellow oil, 440 mg, yield 79%). ^1^H-NMR (CDCl_3_, 400 MHz) δ (ppm) 7.40–7.34 (m, 4H, Ph*H*), 5.23 (q, *J* = 12.3 Hz, 4H, -C*H*_2_PhC*H*_2_-), 3.49 (dd, *J* = 4.1, 2.4 Hz, 2H, -OCOC*H*), 3.01–2.95 (m, 4H, ring C*H*_2_). ^13^C-NMR (100 MHz, CDCl_3_): δ 169.04, 135.53, 129.05, 128.62, 128.38, 128.37, 66.86, 47.30, 46.41. HRMS (ESI): *m*/*z* calcd for C_14_H_14_O_6_ (M^+^) 278.0790; found = 301.0680 ([M + Na]^+^, 100). The NMR spectra of **2a** are shown in the supplementary information.

#### 4.2.2. Synthesis and Characterization Linker of **2b**

2, 3-epoxy-1-propanol (1.56g, 21 mmol) was added to the 50 mL anhydrous DCM solution of 1, 4-dicarboxybenzene (**2a**) (1.66g, 10 mmol), dicyclohexylcarbodiimide (DCC, 4.1 g, 20 mmol) and *N*,*N*-dimethylaminopyridine (DMAP, 27 mg, 2 mmol) in ice-bath at 0 °C for 1 h and then 24 h at room temperature and filtered. The filtrate was evaporated under reduced pressure, and a little ethyl acetate was added. The mixture was maintained at 0 °C for half an hour and filtered. The filtrate was evaporated under reduced pressure to give the crude products which were purified by silica gel column chromatography (silica, ethyl acetate/petroleum ether: *v*/*v* = 1/4) to give product **2b** (white solid, 1.2 g, yield 45%). ^1^H-NMR (CDCl_3_, 400 MHz) δ (ppm) 8.74–7.55 (m, 4H, Ph*H*), 4.70–4.19 (m, 4H, -COOC*H*_2_), 3.37 (s, 2H, ring C*H*), 2.93–2.75 (m, 4H, ring C*H*_2_). ^13^C-NMR (100 MHz, CDCl_3_): δ 165.34, 134.22, 130.96, 130.17, 128.74, 65.91, 49.32, 44.74. HRMS (ESI): *m*/*z* calcd for C_14_H_14_O_6_ (M^+^) 278.0790; found = 301.0679 ([M + Na]^+^, 100). The NMR spectra of **2b** are shown in the supplementary information.

#### 4.2.3. Synthesis and Characterization of Target Polymers **P1**, **P2**

PEI 600 (0.5 mmol) was dissolved in 1 mL anhydrous EtOH, then diglycidyl ether linkers (**2a** or **2b**, 0.5 mmol) were added to the solution. Under the protection of N_2_, the reaction mixture was stirred at 80 °C for 72 h. After the reaction, the mixture was diluted with 2 mL of anhydrous EtOH, and the crude product was precipitated by the addition of anhydrous DCM/cyclohexane (*v*/*v* = 1/1). The precipitate was collected and dried in vacuum to get the product as a pale-yellow oil. The molecular weights of compounds **P1**, **P2** were measured by GPC.

**P1**: Yield 30%. ^1^H-NMR (400 MHz, D_2_O): δ (ppm) 8.16–7.35 (m, 4H, Ph*H*), 4.67–2.15 (m, -C*H*_2_PhC*H*_2_-, -OCOC*H*_2_C*H*-, -NHC*H*_2_C*H*_2_-). GPC: *M_n_* = 17,561, PDI = 1.18.

**P2**: Yield 35%. ^1^H-NMR (400 MHz, D_2_O): δ (ppm) 7.87–7.57 (m, 4H, Ph*H*), 4.42–1.67 (m, -COOC*H*_2_C*H*(OH)C*H*_2_-, -NHC*H*_2_C*H*_2_-). GPC: *M_n_* = 24,550, PDI = 1.87. The ^1^H-NMR spectra of **P1** and **P2** were shown in the supplementary information.

### 4.3. Amplification and Purification of Plasmid DNA

pGL-3 and pEGFP-N1 plasmids were used. The former one as the luciferase reporter gene was transformed in *E. coli* JM109 and the latter one as the enhanced green fluorescent protein gene was transformed in *E. coli* DH5α. Both plasmids were amplified in Luria-Bertani broth at 37 °C and 220 rpm overnight. The plasmids were purified by an EndoFree Tiangen^TM^ Plasmid Kit. Then, the purified plasmids were dissolved in TE (Tris + EDTA) buffer solution and stored at −20 °C. The integrity of plasmids was confirmed by agarose gel electrophoresis. The purity and concentration of plasmids were determined by ultraviolet (UV) absorbance at 260 and 280 nm.

### 4.4. Acid-Base Titration

Briefly, 10 mg of polymers were dissolved in 5 mL of 150 mM NaCl aqueous solution, and 1 N HCl was added to adjust pH to 2.0. Aliquots of 0.1 N NaOH were added, and the solution pH was measured with a pH meter (pHS-25, Shanghai instrument electric science Inc., Shanghai, China) after each addition. For comparison, NaCl (150 mM), PEI 600 and PEI 25 KDa were used under the same experimental conditions. The buffering capacity, defined as the percentage of amine groups becoming deprotonated from pH 5.1 to 7.4, was calculated from equation:

Buffer capacity (%) = 100[(Δ*V*_1_ NaOH – Δ*V*_2_ NaOH) × 0.1 M]/N mol (1)

wherein Δ*V*_1_ NaOH is the volume of NaOH solution (0.1 M) required to bring the pH value of the polymer solution from 5.1 to 7.4, Δ*V*_2_ NaOH is the volume of NaOH solution (0.1 M) required to bring the pH value of the NaCl solution from 5.1 to 7.4, and N mol is the total moles of protonatable amine groups in the polymer.

### 4.5. Polymer Degradation Study

For the polymer degradation study, **P2** (*M_n_* = 24,550) was dissolved in a single-strength (1×) phosphate-buffered saline (PBS) solution and constantly shaken in a 37 °C incubator at 100 rpm. The **P2** solution was withdrawn at different time points and then lyophilized. The relative molecular mass of degraded products was determined by GPC using a Waters 515 pump and a Waters 2410 refractive index detector (25 °C).

### 4.6. Agarose Gel Retardation

Polyplexes at different *w*/*w* ratios (weight ratio of polymer relative to pDNA) were prepared by adding an appropriate volume of the polymer solution to 5 μL of Puc-19 (0.025 mg/mL). The obtained complex solution was then diluted to the total volume of 10 μL. After incubation at 37 °C for 0.5 h, the polyplexes were electrophoresed on a 0.7% (*w*/*v*) agarose gel containing GelRed^TM^ in Tris-acetate (TAE) running buffer at 120 V for 40 min. DNA was then visualized under an ultraviolet lamp by using a Vilber Lourmat imaging system (Paris, France).

### 4.7. Particle Size and Zeta Potential Measurements

Complex size and zeta potential were evaluated by dynamic light scattering (DLS) at 25 °C by Nano-ZS ZEN3600 (Malvern Instruments, Malvern, UK). The polymer/DNA complexes with various *w*/*w* ratios were prepared by adding 1 μg of DNA to the appropriate volume of the polymer solution. The complex solution was then vortexed for 30 s before being incubated at 37 °C for 0.5 h and then diluted up to 1 mL by purity water solution prior to being measured. Data were shown as mean ± standard deviation (SD) based on three independent measurements.

### 4.8. Cell Culture

HEK 293, HepG2, and HeLa cell lines were maintained in DMEM supplemented with 10% (*v*/*v*) heat-inactivated fetal bovine serum, 100 μg/mL streptomycin and 100 IU/mL penicillin at 37 °C in a humidity atmosphere of 5% CO_2_ incubator.

### 4.9. In Vitro Transfection Experiments

Gene transfection of a series of complexes was investigated in HEK 293, HepG2, and HeLa cells. Cells were seeded in 24-well plates (8 × 10^4^ cells/well for HEK 293, 7 × 10^4^ cells/well for HepG2 and HeLa) and grown to reach 70–80% cell confluence at 37 °C for 24 h in 5% CO_2_. Before transfection, the medium was replaced with a serum-free or a 10% serum-containing culture medium containing polymer/DNA (1 μg) complexes at various weight ratios. After 4 h under standard incubator conditions, the medium was replaced with fresh medium containing serum and incubated for another 20 h.

For fluorescent microscopy assays, cells were transfected by complexes containing pEGFP-N1. After 24 h incubation, GFP-expressed cells were observed with an inverted fluorescence microscope (Nikon Eclipse TE 2000E) equipped with a cold Nikon camera. Control transfection was performed in each case using a commercially available transfection reagent PEI 25 KDa and Lipofectamine 2000, based on the standard conditions specified by the manufacture.

For luciferase assays, cells were transfected by complexes containing pGL-3. For a typical assay in a 24-well plate, 24 h post transfection as described above, cells were washed with cold PBS and lysed with 100 μL 1× Lysis reporter buffer (Promega, Madison, WI, USA). The luciferase activity was measured by microplate reader (Model 550, BioRad, Hercules, CA, USA). Protein content of the lysed cell was determined by BCA protein assay. Gene transfection efficiency was expressed as the relative fluorescence intensity per mg protein (RLU/mg protein). All of the experiments were carried out in triplicate.

### 4.10. Cytotoxicity Assay 

The cytotoxicity studies were examined by Cell Counting Kit-8 (CCK-8) assay. Briefly, HepG2 cells were seeded at 0.8 × 10^4^ cells/well in 96-well plates and cultured overnight for 70–80% cell confluence. The medium was replaced with 50 μL of fresh medium, to which 50 μL polyplexes at various concentrations were added to achieve a final volume of 100 μL (0.2 μg DNA/well). Twenty-four hours later, 10 μL CCK-8 mixed in 90 μL PBS was added to each well for an additional 1 h incubation. The absorbance was measured in an ELIEA plate reader (model 550, BioRad, Hercules, CA, USA) at a wavelength of 450 nm. The metabolic activity of the polyplex-treated cells was expressed as relative to untreated cell controls, taken as 100% metabolic activity. Moreover, the cell viability of PEI 25 KDa and Lipofectamine 2000 were also determined.

### 4.11. Statistical Analysis

Data are presented as mean ± standard deviations (±S.D.) of at least three independent samples and each measurement was performed in triplicate. Statistical analysis was determined by analysis of variance tests (ANOVA), using the software of Microsoft Excel 2007. Data sets were compared using two-tailed, unpaired *t*-tests, and a *p* value of <0.05 was considered to be statistically significant.

## 5. Conclusions

A new class of biodegradable poly(amino ester) compounds were synthesized by the ring-opening polymerization between diepoxide and LWM PEI. These materials were applied in the gene delivery as non-viral vectors to study their structure-activity relationships (SAR). The tiny difference of structure largely influenced the buffering capacity, surface properties, and transfection efficiency of the formed polyplexes. Polymer **P2** exhibited higher buffering capacity, proper size, and zeta-potential, which might lead to easier endocytosis and subsequently higher TE. Compared to PEI 25 KDa and Lipofectamine 2000, **P2** could give higher TE and lower cytotoxicity in several cell lines. For example, up to ~2 times higher TE than PEI and Lipofectamine in HEK 293 cells was achieved by employing **P2** as transfection reagent. The lower cytotoxicity of the polymers might come from their biodegradable backbones and hydroxyl groups. Results suggest that such a polymer might be a promising non-viral gene carrier in future in vivo applications. Further modification of the polymer structure and relative mechanism studies are now in progress.

## Figures and Tables

**Table 1:** The buffer capacity of newly synthesized polymers and polyethylenimine (PEI).

Polymer	PEI 600	PEI 25K	P1	P2
Buffering capacity (%)	11.20	18.09	25.85	28.37

## Supplementary Materials

Supplementary materials are available online.
