# Supplementary material for: Biodegradable Poly(Amino Ester) with Aromatic Backbone as Efficient Nonviral Gene Delivery Vectors
Source: Molecules. 2017 Mar 31;22(4):566. doi: 10.3390/molecules22040566 (PMC6154102; doi:10.3390/molecules22040566)

Supplementary Information: Biodegradable poly(amino ester) with aromatic backbone as efficient nonviral gene delivery vectors

Qiang Liu, Rong-Chuan Su, Wen-Jing Yi * and Zhi-Gang Zhao *

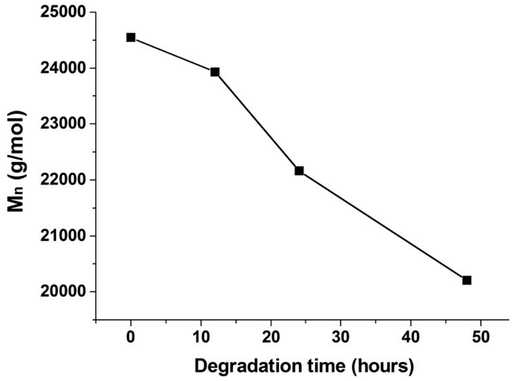

Figure S1. Change of molecular weight of **P2** (M_n = 24,550 Da) with degradation time in PBS (pH = 7.4).

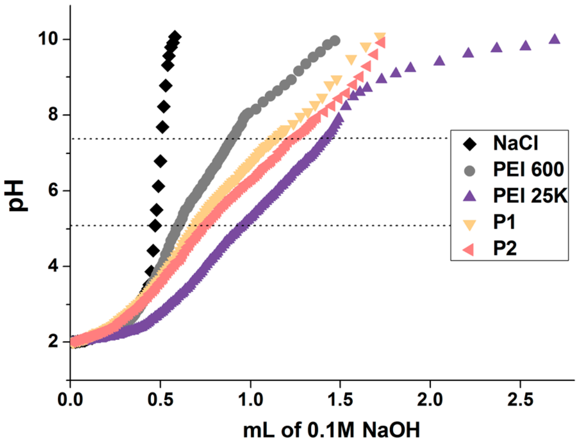

Figure S2. Acid-base titration profiles of **P1**, **P2** and PEI.

Spectra

a. ^{1}H-NMR Spectra

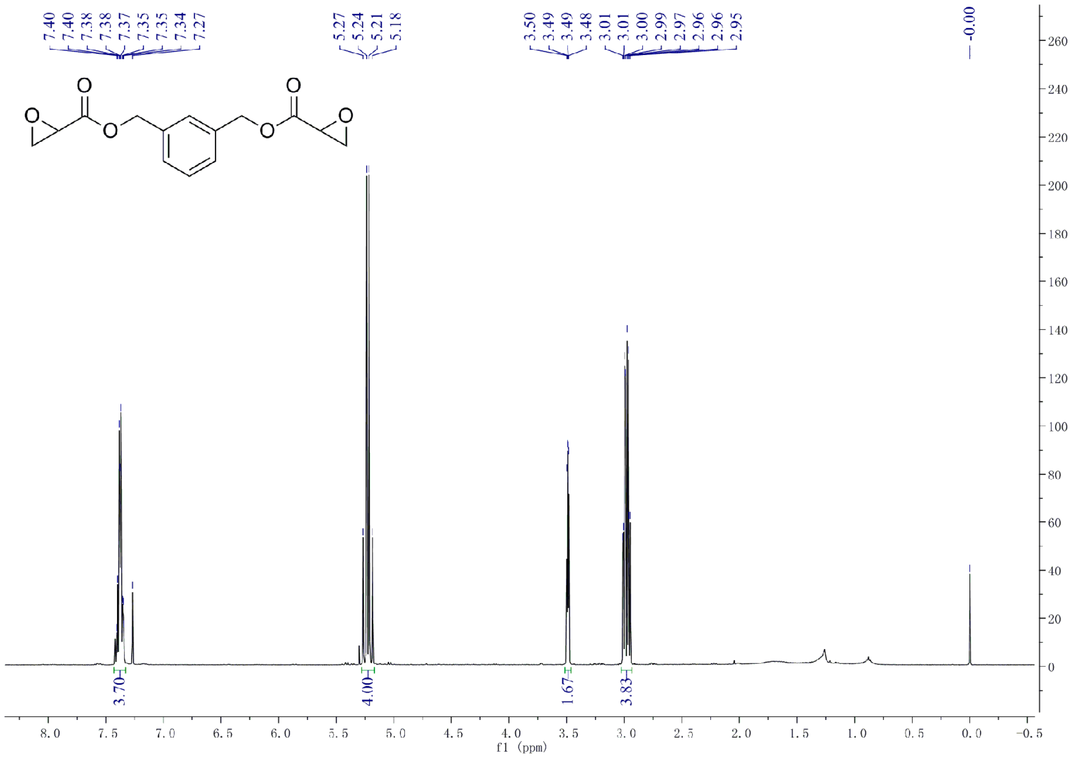

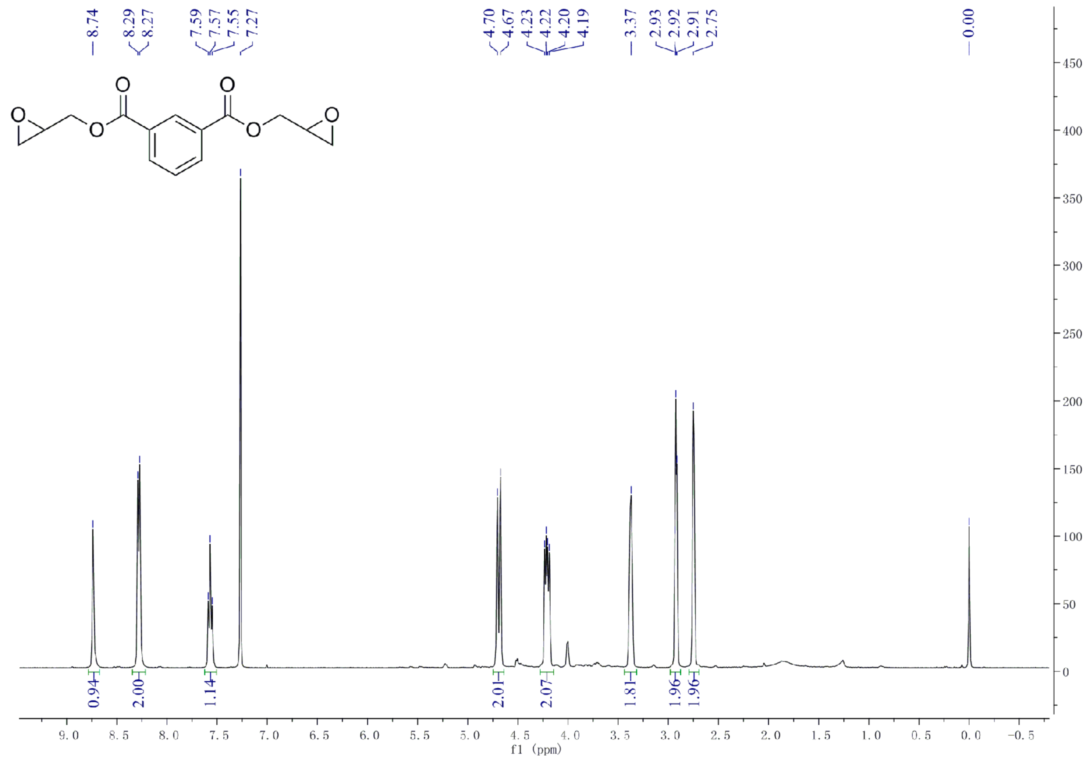

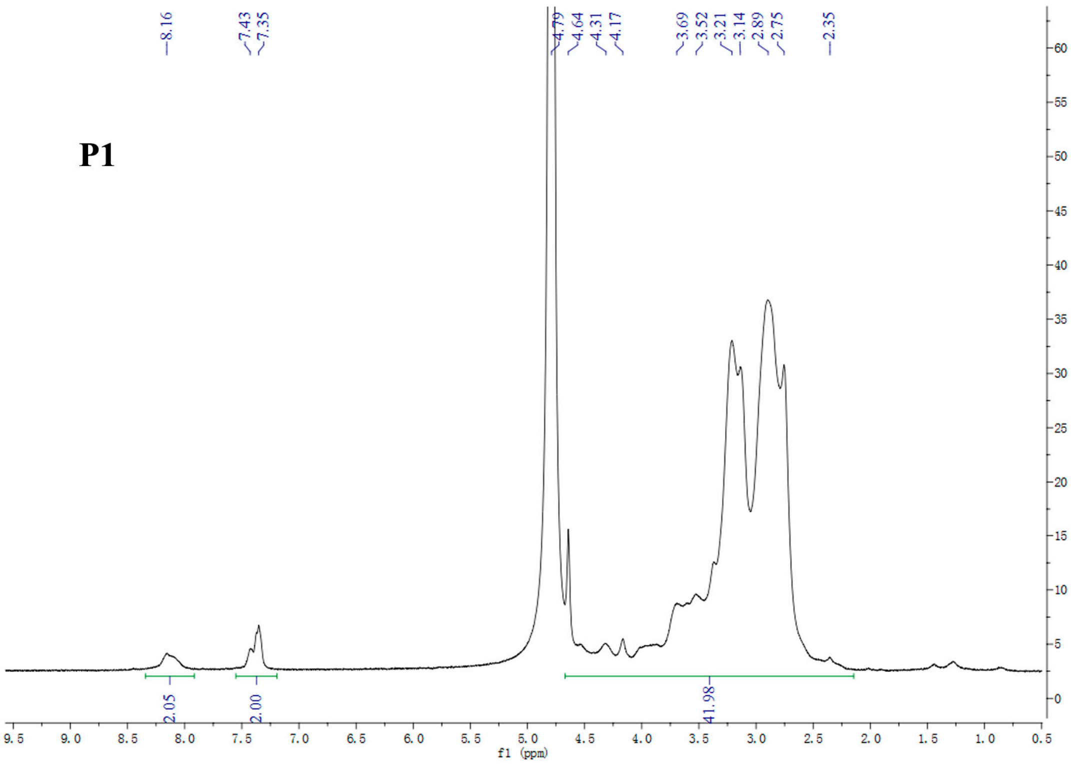
P1
8.16
7.43
7.35
4.79
4.64
4.31
4.17
3.69
3.52
3.21
3.14
2.89
2.75
2.35
2.05
2.00
41.98
f1 (ppm)

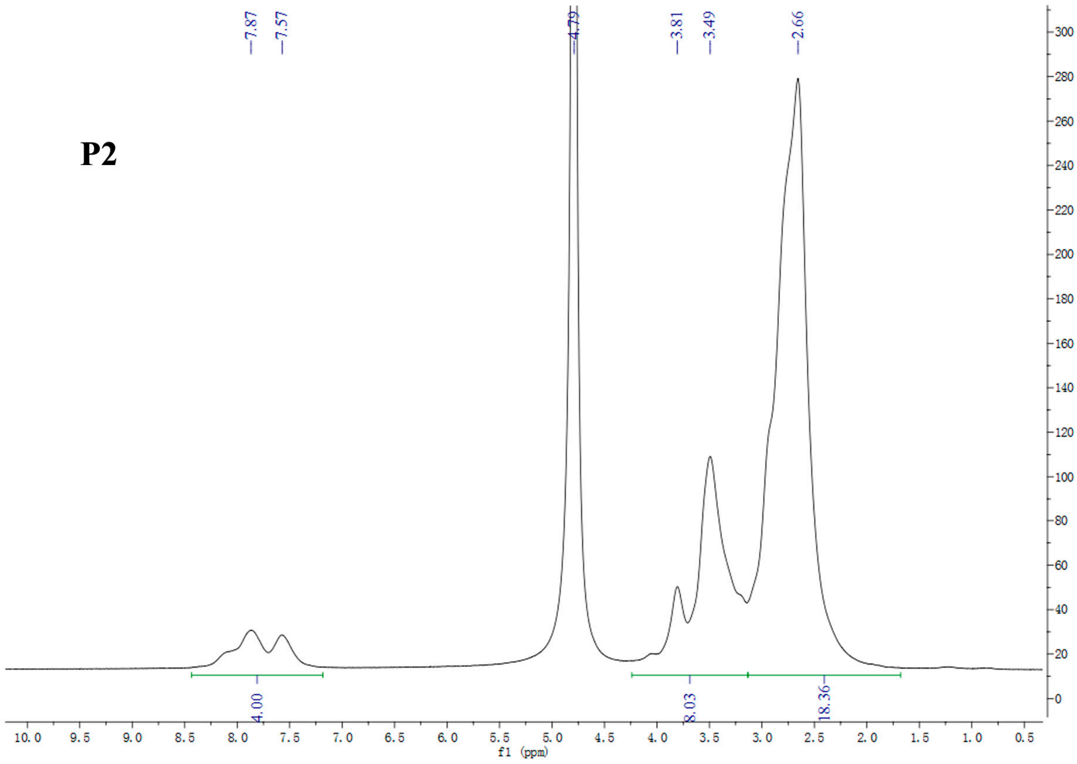
P2
7.87
7.57
4.79
3.81
3.49
2.66
4.00
8.03
18.36
f1 (ppm)

b. **^{13}C-NMR Spectra**

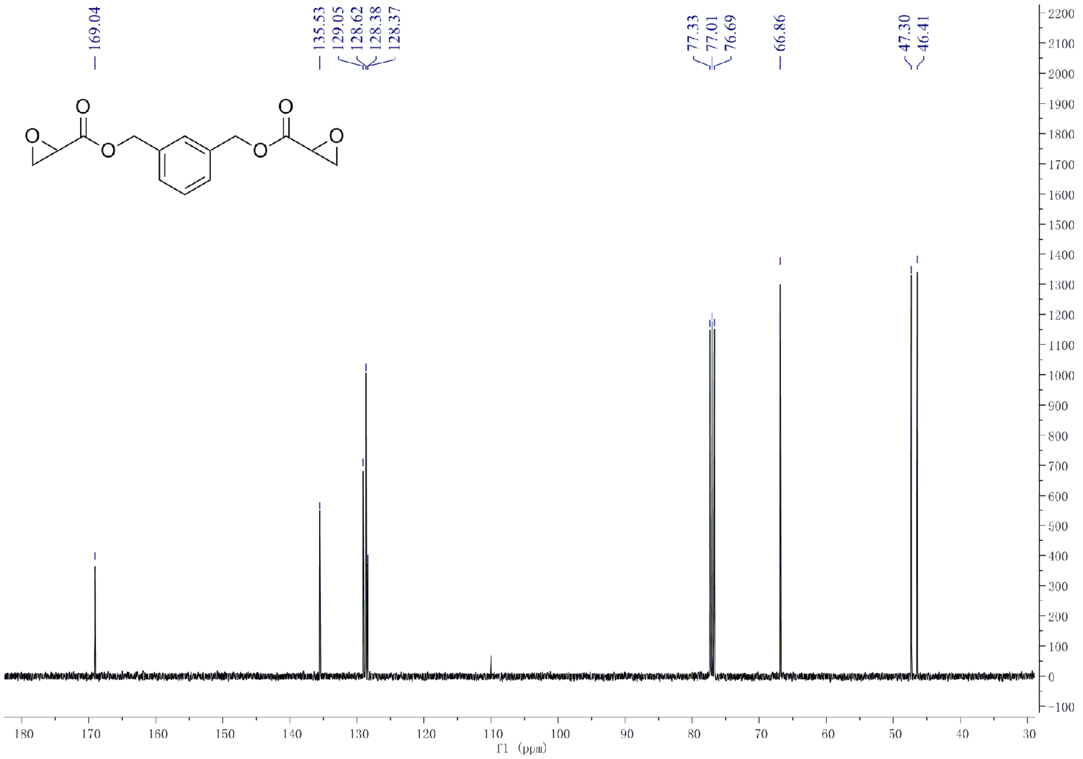

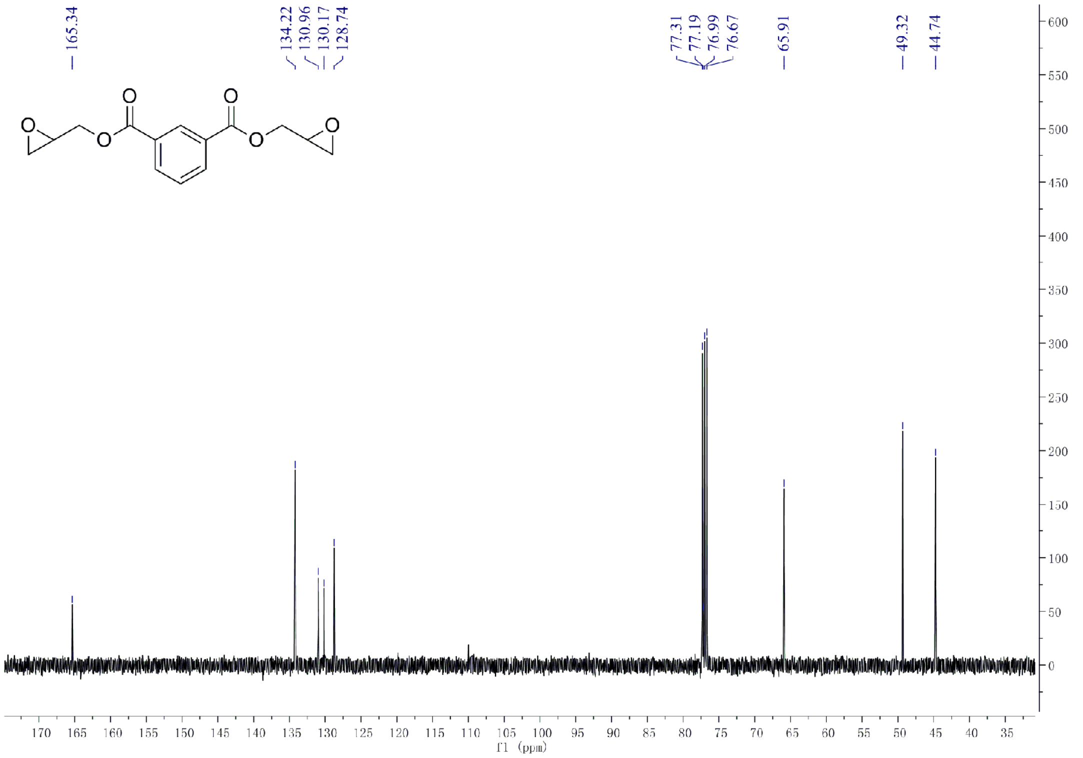

Supplement: Supplementary file 1 [file molecules-22-00566-s001.pdf]
